# The Impact of Social Stress on Trustworthiness Judgments in Schizophrenia

**DOI:** 10.1002/pchj.816

**Published:** 2024-12-08

**Authors:** Natália Čavojská, Vladimír Ivančík, Alexandra Straková, Jakub Januška, Daniel Dančík, Barbora Vašečková, Ľubica Forgáčová, Dana Krajčovičová, Jakub Kraus, Ján Pečeňák, Anton Heretik, Michal Hajdúk

**Affiliations:** ^1^ Department of Psychiatry, Faculty of Medicine Comenius University in Bratislava Bratislava Slovakia; ^2^ The Centre for Psychiatric Disorders Research, Science Park Comenius University in Bratislava Slovakia; ^3^ Department of Psychology, Faculty of Arts Comenius University in Bratislava Slovakia; ^4^ Department of Psychiatry Slovak Medical University in Bratislava Slovakia

**Keywords:** facial trustworthiness, schizophrenia, social stress, trust

## Abstract

The present study examines the impact of induced social stress on facial trustworthiness judgments in individuals with schizophrenia (SCZ) and the associations between symptoms and trustworthiness ratings. Thirty‐three individuals with SCZ and forty healthy controls (HC) were asked to rate the trustworthiness of 24 digitally morphed faces in two counterbalanced conditions. Mild social stress was induced by listening to loud noises from a busy street. BPRS, CAPE‐42, and the Paranoia Scale measured the severity of symptoms. We did not observe significant differences in trustworthiness judgments between the patient and control groups. Social stress did not impact trust judgments. Paranoia was negatively connected to trustworthiness ratings in the control group. Subjective rating of stress in noise condition was negatively associated with paranoia only in the control sample. In the patient group, a negative correlation was found between trustworthiness ratings in stress conditions and the severity of self‐reported negative symptoms. Our results suggest that mild social stress does not significantly affect trustworthiness judgments in either patients with SCZ or in HC. Differences between the patient and control groups in trustworthiness judgments were negligible. Overall, the results of this study can be considered mainly negative and contrast with previous studies. The stress induction paradigm or a smaller sample size might cause observed results. In controls, biased face perception was linked to trait paranoia. By contrast, in SCZ, other factors might impact trust perception and need further examination.

## 1 Introduction

Individuals with schizophrenia (SCZ) exhibit various social–cognitive biases, such as increased attention to threatening stimuli (Bentall et al. 2001), personalizing bias (Kinderman and Bentall 1996), bias toward intentionality (Buck et al. 2018), and hostility bias (Combs et al. 2007). These biases are primarily associated with positive symptoms, specifically paranoia and its more severe form, persecutory delusions (Couture et al. 2010; Kirk et al. 2013; Pinkham, Harvey, and Penn 2016). The central aspect of persecutory delusions is the unfounded belief that others might cause harm (Freeman 2016; Freeman and Garety 2000). Therefore, threat anticipation and distorted processing of socially salient stimuli are central to understanding paranoia (Green and Phillips 2004).

People spontaneously evaluate the faces of others in several dimensions, such as attractiveness, sociability, dominance, aggressivity, or trustworthiness (Engell, Haxby, and Todorov 2007; Oosterhof and Todorov 2008; Todorov, Baron, and Oosterhof 2008). Trustworthiness judgments are particularly vital as they enable quick inferences about potential social threats and harmful intentions of others (Willis and Todorov 2006). The study of trustworthiness judgments in SCZ has recently gained substantial attention. However, current studies have provided mixed findings. Three studies found that patients perceive faces as less trustworthy than controls (Hooker et al. 2011; Pinkham, Hopfinger, and Penn 2012; Pinkham, Harvey, and Penn 2016), while others showed the opposite pattern: patients perceived faces as more trustworthy than controls (Baas et al. 2008; Couture et al. 2010). Many studies found no differences (Hajdúk, Krajčovičová, et al. 2019; Haut and MacDonald 2010; Trémeau et al. 2016; Woolley et al. 2017). Despite this heterogeneity, most studies (e.g., Couture et al. 2010; Haut and MacDonald 2010; Pinkham, Harvey, and Penn 2016) identified associations between trust ratings and the severity of trait paranoia or persecutory delusions. This might suggest that the trustworthiness bias is more pronounced during the acute phase of the illness when positive symptoms are more prominent.

Rapid trustworthiness judgments are typically based on limited knowledge or experience with a person and under time pressure. Therefore, we can expect that situational characteristics, especially social stress or an immediate threat, might negatively impact or bias these judgments.

SCZ is characterized by elevated stress sensitivity that is linked to threat anticipation (Reininghaus et al. 2016). Given the heightened sensitivity of individuals with SCZ to stressors, such as noise, it is plausible that noise could further skew the already biased social–cognitive processes in individuals with SCZ. SCZ shows a marked increase in incidence among urban dwellers (McGrath et al. 2004; van Os, Kenis, and Rutten 2010). Possible mediators may include heightened exposure to social stress through overcrowding or environmental pollutants (such as noise) (Lederbogen, Haddad, and Meyer‐Lindenberg 2013). Noise exposure is an environmental stressor that stimulates the endocrine and autonomic nervous systems, increasing catecholamine and cortisol levels (Clark and Paunovic 2018).

In a study conducted by Freeman et al. (2015), patients with persecutory delusions were randomized into two conditions: exposure to a busy urban environment versus remaining inside. Exposure to busy streets was associated with increased paranoia, a response mediated by anxiety, depression, and negative beliefs. This suggests that an increase in negative affect could form a pathway through which social exposure in urban environments triggers paranoid thoughts. Similarly, Ellett, Freeman, and Garety (2008) found that going outside (buying newspapers on a busy street) versus performing relaxation tasks indoors led to an increase in paranoia, anxiety, negative beliefs about others, and jumping to conclusions. A possible explanation of how urban environments influence mental health is through social defeat—individuals living in urban areas are exposed to higher levels of social competition, leading to social defeat stress (Selten and Cantor‐Graae 2005).

In healthy individuals, social judgments are influenced by the current affective states (Forgas 1994, 1995). For example, higher stress and consequent negative affect or anxiety during stressful situations may negatively bias face perception in healthy adults (Dyer et al. 2022; Forgas 1994, 1995). There is also a growing interest in understanding the effect of situational social stress on social cognitive abilities in SCZ or broadly on the psychosis continuum. For instance, in individuals with higher vulnerability to psychosis, social stress can lead to more monocausal attribution (i.e., the tendency to attribute potential causes of events to a single source rather than considering multiple factors) (Randjbar et al. 2011) and a higher tendency to jump to conclusions (Lincoln et al. 2010; Moritz et al. 2015). In contrast, Köther, Lincoln, and Moritz (2018), in a large sample of patients, relatives, and individuals with attenuated positive symptoms, did not find a direct negative impact of social and acoustic stress on emotion recognition.

Furthermore, Urbańska, Moritz, and Gawęda (2019) demonstrated that social stress in individuals with SCZ leads to a higher conviction in paranoid beliefs and a slight decrease in mentalizing accuracy. The studies often used higher‐order social cognitive measures, such as mentalizing tasks or emotion recognition, which tap into only a limited aspect of the broad construct of social cognition. The impact of social stress in such cases appears to be limited. The adverse effects of stress might be more subtle and present in tasks that rely more on automatic processing, such as making quick, trustworthy judgments. This assumption was supported by Pinkham, Hopfinger, and Penn (2012), who found that individuals with SCZ perceived faces as less trustworthy during magnetic resonance scanning than when outside of the scanner.

Taken together, contextual features that induce stress may modulate trustworthiness judgments in individuals with SCZ. Stressful contexts might trigger negative trustworthiness judgments, particularly in highly paranoid individuals (Pinkham, Hopfinger, and Penn 2012). Therefore, this study aims to test the impact of induced social stress (exposure to environmental noise of busy, crowded streets with indistinguishable babble) on trustworthiness judgments in individuals with SCZ. First, we hypothesize that social stress will lead to faces being perceived as less trustworthy. Second, we hypothesize that elevated paranoia or the presence of persecutory delusions will be associated with the perception of faces as less trustworthy.

## 2 Methods

### 2.1 Sample

The final sample consisted of 33 participants (57.57% male) with SCZ spectrum disorders recruited from the Psychiatric Clinic of University Hospital Bratislava and outpatient psychiatric practices in Bratislava. Participants with SCZ had a mean age of 36.55 years (SD = 9.08), a mean of 14.73 years of education (SD = 2.58), and a mean illness duration of 11.79 years (SD = 7.95). The healthy control (HC) sample consisted of 40 individuals (45% male) from the local community. HC had a mean age of 30.76 years (SD = 10.38) and a mean of 15.45 years of education (SD = 3.26). Diagnoses were confirmed or ruled out by the Mini International Neuropsychiatric Interview (Sheehan et al. 1998) administered by a trained Ph.D. student. General exclusion criteria for both groups included the presence of serious untreated somatic illness, neurological illness, severe head injury or a history of epileptic seizure, moderate to severe substance use disorder, and uncorrected vision. The research project was approved by the Ethical Review Board of University Hospital Bratislava and the Medical Faculty of Comenius University in Bratislava. All participants provided written informed consent. Participants received a voucher worth €20 for their participation in the study.

This study was conducted as part of a larger project. We did not specifically sample individuals based on an a priori‐defined sample size estimated for the Trustworthiness Judgments Task. The sample size was primarily determined by resource constraints, including the availability of participants and the project duration (Lakens 2022). The task structure differs from those used in previous studies, so relying on prior studies for effect size estimation may be misleading.

### 2.2 Measures

#### 2.2.1 Trustworthiness Judgment Task

**FIGURE 1:**
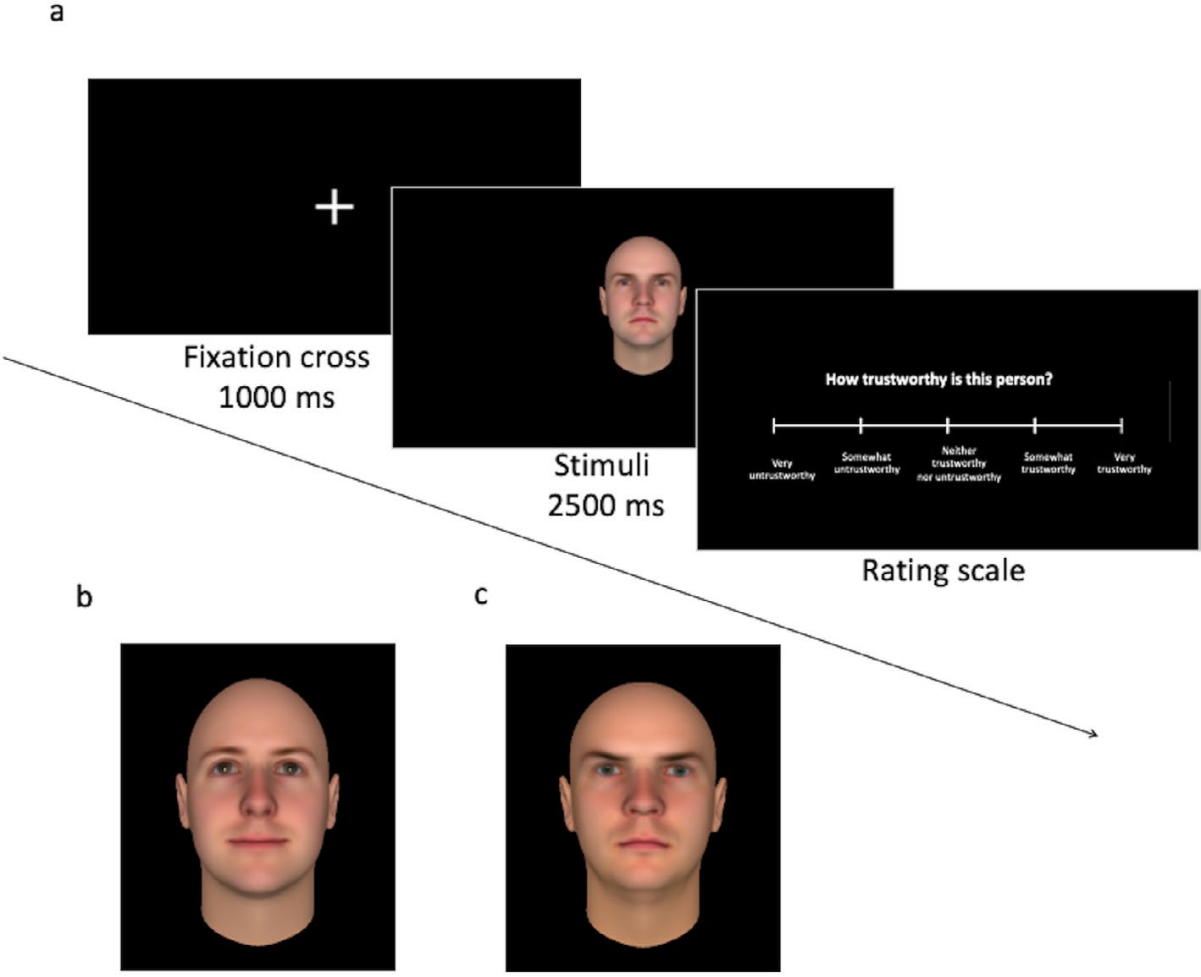
Trustworthiness task—(a) overview of the task setup, (b) example of a very trustworthy face, and (c) example of a very untrustworthy face. Images were used from the Todorov Lab Database (Oosterhof and Todorov 2008).

Stimuli were drawn from the Todorov Lab Database (Oosterhof and Todorov 2008; Todorov, Baron, and Oosterhof 2008). We created two independent sets of faces, each containing 24 images of Caucasian male faces with emotionally neutral expressions but digitally morphed facial attributes (e.g., face width) to manipulate the trustworthiness of presented faces: 12 extremely trustworthy and 12 extremely untrustworthy faces. Both sets were matched on trustworthiness levels according to the face perception model (Oosterhof and Todorov 2008). The faces were presented on a black background for 2500 ms. A fixation cross was shown for 1000 ms before the stimuli. Faces in each set were randomized but presented in a fixed order to all participants. Following each stimulus, a 5‐point Likert‐type rating scale was presented [ranging from −2 (*very untrustworthy*) to 2 (*very trustworthy*)]. Participants were asked to evaluate how trustworthy or untrustworthy they considered each presented face (Figure 1).

Additionally, participants completed the trustworthiness task in the counterbalanced order (normal vs. noise). Some studies (e.g., Ellett, Freeman, and Garety 2008; Freeman et al. 2015) assessed paranoia in real‐world environments containing threats of interpersonal harm, such as exposure to a busy street. We used a sound recording of noise from a busy city street (featuring the sound of traffic, crying, shouting, and indistinguishable babble) at approximately 80 dB, similar to those used by Urbańska, Moritz, and Gawęda (2019), Wright et al. (2014), and Lincoln et al. (2015), presented through headphones to induce psychosocial stress. Participants were asked to rate subjective stress during the face presentation after each experimental condition on a 10 cm visual analog scale, where higher scores indicated more pleasant feelings (lower stress).

#### 2.2.2 Psychopathology

##### 2.2.2.1 Brief Psychiatric Rating Scale (BPRS)

Psychopathology was assessed using the BPRS—UCLA version (Ventura et al. 1993). Items were classified into four factors (positive symptoms, agitation/mania, negative symptoms, and depression/anxiety) based on the factor analytic study by Kopelowicz et al. (2008). Trained Ph.D. students administered the scale, and ratings were reviewed in consultation with a senior clinician (M.H.) with extensive BPRS experience.

##### 2.2.2.2 Community Assessment of Psychic Experiences (CAPE 42)

The CAPE 42 (Stefanis et al. 2002) measures psychotic experiences in the general population. This questionnaire contains 42 items assessing the frequency and associated distress of psychotic experiences using a 4‐point Likert scale. It is divided into three subscales: positive, negative, and depression. Mean scores were calculated for each dimension, with higher scores indicating greater severity of that symptom domain. In the present study, we only included results from the frequency subscale.

##### 2.2.2.3 Paranoia Scale

The Paranoia Scale (Fenigstein and Vanable 1992) is a 20‐item self‐reported measure designed to assess paranoid ideation. Participants respond on a 5‐point Likert scale. The items address mistrust, interpersonal sensitivity, and persecutory ideation. Higher scores indicate higher levels of paranoid ideation. We calculated the mean item score for analysis.

### 2.3 Statistical Analyses

All statistical analyses were performed using the software JASP (version 0.16). For trustworthiness judgments and manipulation checks (differences in pleasantness feelings), we utilized repeated measures ANOVA with condition type (normal vs. noise) as a within‐subjects factor and group (SCZ vs. HC) as a between‐subjects factor. In addition, the Pearson correlation coefficient was used to estimate the strength of associations between variables.

## 3 Results

### 3.1 Demographic Characteristics and Descriptive Statistics

Demographic and clinical characteristics are shown in Table 1. On average, patients were older than controls (*t*(71) = −2.501, *p =* 0.015, *d* = 0.588), but samples were matched by years of education (*t*(71) = 1.035, *p =* 0.304, *d* = 0.243). The mean duration of illness was *M* = 11.79, SD = 7.95, and the mean number of hospitalizations was *M* = 3.56, SD = 3.12. There were no significant differences in the proportion of males and females across groups (*χ* ^2^(1) = 1.144, *p* = 0.285, *V* = 0.125).

**TABLE 1:** Demographic and clinical variables.

	SCZ		HC		
	N/M/%	SD	N/M/%	SD	*t*/*χ* ^2^
Gender					
Male (%)	57.57		45.00		*χ* ^2^ = 1.144, *p* = 0.285
Age	36.55	9.08	30.76	10.38	*t* = −2.50, *p* = 0.015
Years of education	14.73	2.58	15.45	3.26	*t* = 1.04, *p* = 0.304
Duration of illness	11.79	7.95			
Psychopathology					
BRPS—positive	1.30	0.38			
BPRS—negative	1.81	0.87			
BPRS—depression/anxiety	1.67	0.85			
BPRS—agitation/mania	1.11	0.20			
Paranoia scale	1.97	0.73	1.76	0.55	*t* = −1.368, *p* = 0.175

Abbreviations: BPRS, Brief Psychiatric Rating Scale; HC, healthy controls; SCZ, Schizophrenia; *t*, test; *χ* ^2^, Chi square.

### 3.2 Manipulation Check for the Stress Condition

**FIGURE 2:**
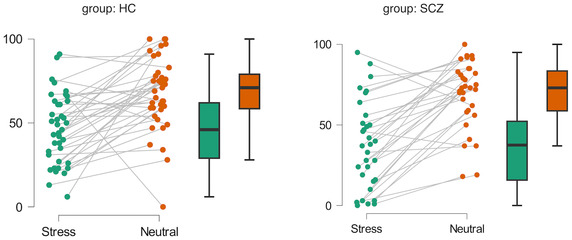
Differences in subjective stress across conditions separately for SCZ and HC.

To test whether loud noises increase stress, we utilized repeated measures ANOVA with condition (normal vs. noise) as the within‐subject factor and group (HC vs. SCZ) as the between‐subject factor. We found a significant effect of condition on subjective stress (*F*(1, 69) = 70.554, *p* < 0.001, *η* = 0.256). However, the effect of group (*F*(1, 69) = 0.692, *p* = 0.408, *η* = 0.005) and the interaction between condition and group (*F*(1, 69) = 2.514, *p* = 0.117, *η* = 0.009) were not significant. These results confirm that our manipulation check demonstrated that noises cause an increase in subjective stress (Figure 2).

### 3.3 Impact of Social Stress on Trustworthiness Judgments

**FIGURE 3:**
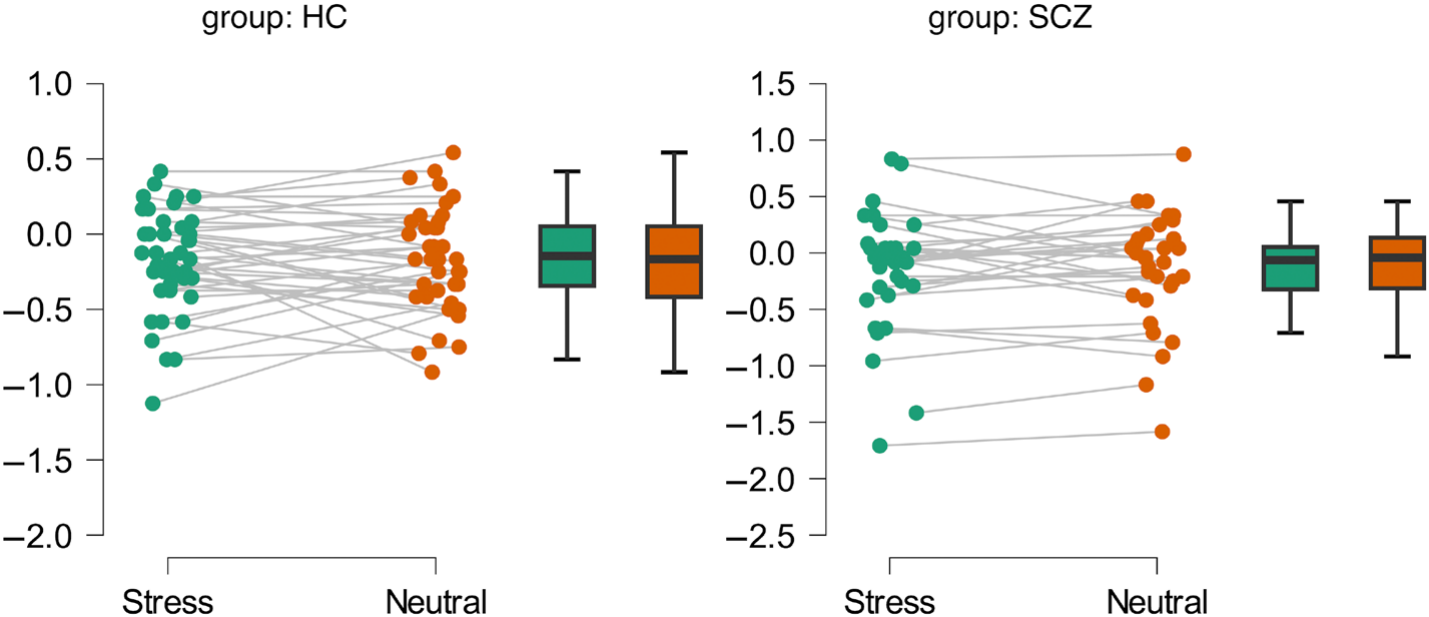
Trustworthiness ratings across conditions in both groups.

We did not find a significant effect of condition (normal vs. noise) on trustworthiness judgments (*F*(1, 70) = 0.042, *p* = 0.838, *η* < 0.001). Similarly, the effects of group (*F*(1, 70) = 0.119, *p* = 0.731, *η* = 0.002) and interaction between condition and group on trustworthiness ratings (*F*(1, 70) = 0.087, *p* = 0.769, *η* < 0.001) were also not significant (Figure 3).

### 3.4 Trustworthiness and Paranoia Severity

We analyzed results separately for patients and HC. In controls, we found that more severe paranoia measured by the Paranoia Scale was negatively associated with trustworthiness ratings in both stress (*r* = −0.462, *p* = 0.006) and control condition (*r* = −0.353, *p* = 0.025). Subjective stress ratings in stress conditions were negatively associated with paranoia (*r* = −0.384, *p* = 0.016). In the patient group, the trustworthiness task was unrelated to the severity of paranoia as measured by the Paranoia Scale and positive symptoms measured by BPRS. The suspiciousness item from the BPRS was not significantly related to trustworthiness ratings (*r*_neutral_ = −0.056 and *r*_stress_ = −0.157).

### 3.5 Exploratory Analysis of Other Symptoms Domains

In the exploratory part of the analysis, we found that in controls, the CAPE‐42 subscales were not associated with any trustworthiness task parameters. On the other hand, in patients, we observed a moderate negative correlation between trustworthiness ratings in stress conditions and the severity of self‐reported negative symptoms measured by CAPE‐42 (*r* = −0.378, *p* = 0.036). These results should be interpreted with caution due to the exploratory nature of the analysis.

## 4 Discussion

The present study examined the impact of mild social stress on trustworthiness judgments in individuals with SCZ. We did not find differences in the mean trustworthiness judgments between the patient and the control group. This result aligns with previous studies such as Hajdúk, Krajčovičová et al. (2019), Haut and MacDonald (2010), Trémeau et al. (2016), and Woolley et al. (2017), which also did not find this bias in stabilized patients with SCZ. As outlined in the introduction, some studies showed differences, but based on the largest study (Pinkham et al. 2016), we can expect a small effect (*d* = 0.27), and therefore, extensive samples are needed to have sufficient statistical power. The consistency across these studies with stabilized patients could be attributed to similarities in the stability of patient conditions, where symptoms are managed to a degree that diminishes the variability in trustworthiness judgments. It is also worth considering whether the methods used to assess trustworthiness, which often rely on subjective interpretations of facial expressions, are sensitive enough to detect subtle differences.

We also did not find differences across conditions, suggesting that our experimental stress induction did not impact the perception of faces, even though it was subjectively rated as stressful by both groups. It seems that our stress induction was not sufficient and did not have any substantial impact on controls and stabilized patients. Our results contrast with earlier studies that suggested altered social cognition under stress. For instance, Pinkham, Hopfinger, and Penn (2012) found that individuals with SCZ may perceive faces as less trustworthy when exposed to stress, likely due to the heightened threat sensitivity commonly observed in SCZ. Furthermore, previous studies (Ellett, Freeman, and Garety 2008; Freeman et al. 2015) demonstrated that walking in a busy immersive environment increased paranoia and negative affect.

Additionally, social stress, operationalized as exposure to people with hostile facial expressions, triggered paranoia (Pot‐Kolder et al. 2018). Some studies (Lincoln et al. 2009; Moritz et al. 2011) have also shown the impact of noise exposure (similar to recording the noise we used) on the increase of paranoid beliefs. The discrepancy between these findings and our own could stem from variations in the stress induction techniques. Our study utilized a milder form of stress (recording of a noisy street), which, although subjectively acknowledged by participants (similar to the findings of Lincoln et al. 2015, who observed both self‐reported stress and physiological changes following exposure to recorded street noise), may not be fully analogous to real social situations and might not have been sufficient to alter social cognitive processes. Further development and validation of social stress induction in psychosis are needed.

Our results only partially supported the role of psychosocial stress in paranoia, as only HC with higher paranoid thoughts perceived stress conditions more negatively. Surprisingly, this pattern was not observed in the clinical sample. Several factors might explain this discrepancy. Paranoia in HC might be considered more of a stable trait disposition, in contrast to patients among whom the severity of paranoia or persecutory delusions may be more dynamic and context dependent (Fan et al. 2022). In patients, other factors such as motivational dysfunction as part of negative symptoms or cognitive impairment might also modulate trustworthiness judgments. Some studies found that patients perceived faces as more trustworthy than controls. This suggests heterogeneity in trustworthiness bias in patients. Future studies on larger samples might classify patients based on the prominence of positive or negative symptoms or the severity of cognitive impairment and investigate which clinical characteristics are associated with more severe trustworthiness bias.

Additionally, the chronic nature of paranoia (trait paranoia) in some individuals with SCZ could modulate every day trust judgments differently than acute experimental manipulations. Future studies might explore how trait/state paranoia levels are linked to social stress and social cognitive biases. The results may have been impacted by the scale we used. The Paranoia Scale uses only a single score that averages all aspects of paranoia. Novel measures, such as the revised version of Green et al.'s Paranoid Thought Scale (Freeman et al. 2021), enable measurement of ideas of reference and ideas of persecution that might be affected differently by social stress induction and could highlight other meaningful group differences.

In healthy individuals, stable beliefs that people cannot be trusted might lead to aberrant processing all faces as less trustworthy, subsequently leading to problems in interpersonal functioning (Hajdúk, Klein, et al. 2019). Our study revealed that patients who reported more severe negative symptoms perceived faces during stressful conditions as less trustworthy. This observation aligns with the notion that negative symptoms, specifically social withdrawal, social anhedonia, or avolition, are closely linked to the observed difficulties in social functioning (Strauss et al. 2013). One possible explanation for the relationship between negative symptoms and trust perception could be aberrant social reward processing (Catalano, Heerey, and Gold 2018; Lee et al. 2019). These patients might perceive faces as less trustworthy due to insufficient perceived rewards from social interactions. This might suggest that trust judgments are linked to two broad social motivation tendencies: heightened avoidance (related to paranoia) and low social approach (associated with negative symptoms).

Several limitations of the present study should be acknowledged. First, our patients were clinically stabilized, showing only minor levels of positive symptoms, which could limit the generalizability of our findings to patients with acute psychosis. Due to lower variability in paranoia, both on the Paranoia Scale and in BPRS suspiciousness, correlation coefficients might be attenuated. Using generated facial stimuli based on the validated algorithm also poses a limitation. While this approach has advantages, such as the opportunity to manipulate each facial feature, these may not evoke the same responses as authentic human faces during real social interactions. Additionally, all the images displayed Caucasian males without hair. Therefore, future studies might benefit from employing more diverse and realistic stimuli (e.g., photographs of real faces). The nature of our stress induction procedure might be too mild, suggesting the need for employing more robust techniques. It is crucial to consider the intensity and duration of the stress induction method, whether our “social noise” was analogous to a social situation, and the baseline stress levels of participants. Future research might employ different stress induction methods, such as the Trier social stress test (Kirschbaum, Pirke, and Hellhammer 1993), which is considered a gold standard in experimental stress research and includes social–evaluative threat and an element of uncontrollability (Dickerson and Kemeny 2004). Studies on healthy participants show that this procedure impacted the perception of faces perceived as less trustworthy (Toet, Bijlsma, and Brouwer 2017). Moreover, HC were younger than patients but matched in years of education. The correlation part of the study was more exploratory and needs to be replicated on a larger sample to see how robust the findings are. The last but significant limitation of the current study is a small sample size that might lead to low statistical power to detect small effects, which might be even more pronounced in combination with insufficient stress induction. As noted earlier, only small differences can be expected in stabilized patients.

In future research, trustworthiness ratings could be linked to physiological measures of the autonomic nervous system, such as skin conductance response, heart rate, or pupil reactivity, to assess stress reactivity better. Additionally, analyzing saliva samples could help to detect short‐term cortisol fluctuations as an objective measure of stress response (Dickerson and Kemeny 2004). Examining a combination of psychological and physiological variables may provide a more comprehensive view of the nature of social processes (also in the context of paranoia). It may help elucidate physiological correlates of impairments in social stimuli processing (Frost‐Karlsson et al. 2019). Furthermore, eye‐tracking could help determine whether social stress impacts higher‐order inferential processes or whether its effects are more pronounced on low‐level visual attention processes (different scan paths depending on the condition).

## 5 Conclusion

In sum, the present study did not find a negative effect of stress on facial trustworthiness judgments in individuals with SCZ or HC. Our experimental stress induction was successful but not strong enough to bias facial judgments. Trustworthiness judgments were related to the severity of paranoia but, contrary to previous findings, only in a sample of HC. The study provided new insights into the potential role of negative symptoms in facial trustworthiness judgments in SCZ. Overall, the results of this study can be considered predominantly negative and in contrast with previous studies. These unexpected findings prompt further investigation into the effects of stress on different SCZ populations, potentially informing both clinical practices and therapeutic interventions. Studies should also focus on developing and validating new paradigms for social stress induction.

## Conflicts of Interest

The authors declare no conflicts of interest.
